# Flexible low-voltage organic transistors with a transit frequency of 40 MHz and an on/off current ratio of 10 orders of magnitude

**DOI:** 10.1126/sciadv.aeb9693

**Published:** 2025-12-17

**Authors:** Ute Zschieschang, Hagen Klauk

**Affiliations:** Max Planck Institute for Solid State Research, Heisenbergstr. 1, 70569 Stuttgart, Germany.

## Abstract

Organic thin-film transistors (TFTs) are of interest for flexible displays, sensors, and circuits. For all these applications, the TFTs must have a large on/off current ratio and a high transit frequency. A major challenge is to simultaneously maximize the on/off ratio and the transit frequency, because a high transit frequency requires a small channel length, which often leads to short-channel effects, including a large off-state drain current. Here, we lift this fundamental compromise and demonstrate organic TFTs that exhibit a complete absence of detrimental short-channel effects for channel lengths down to 300 nm, making it possible to simultaneously achieve the largest on/off current ratio (10^10^) and the highest transit frequency (40 MHz) reported to date for flexible organic TFTs, despite the fact that these TFTs operate at very low voltages (≤3 V). These organic TFTs therefore meet the fundamental static and dynamic performance requirements for flexible, high-frequency, low-voltage, low-power electronic systems.

## Teaser

Flexible organic transistors with an on/off ratio of 10 orders of magnitude and a transit frequency of 40 MHz are reported.

## INTRODUCTION

Organic thin-film transistors (TFTs) are field-effect transistors in which the semiconductor is a thin layer of conjugated organic molecules. The main incentive for the development of organic TFTs is the fact that they can be fabricated at relatively low process temperatures (<200°C) and therefore not only on glass but also on flexible polymeric substrates. This makes organic TFTs particularly useful for flexible active-matrix displays (*1*), sensor arrays (*2*), and integrated circuits (*3*, *4*). In essentially all applications for which organic TFTs are being envisioned, the TFTs must have both a large on/off current ratio and a high transit frequency. The on/off current ratio is a static performance parameter and defined as the ratio between the on-state drain current and the off-state drain current, while the transit frequency is a dynamic performance parameter and defined as the highest frequency at which the transistors are able to switch or amplify electrical signals. For example, TFTs used in active-matrix displays must have a large on-state drain current and a high transit frequency to facilitate rapid charging and discharging of the pixel capacitors, and they must have a small off-state drain current to minimize charge leakage and cross-talk (*5*). TFTs used in sensors, memories, and digital circuits must have a large on/off ratio to accurately and reliably discriminate between a large number of discrete signal levels (*6*), and they must have a high transit frequency to minimize signal delays and maximize data rates (*7*). In analog circuits, a large on/off ratio provides improved signal amplification, while a high transit frequency is important to achieve a large bandwidth (*8*). For all applications, in particular for mobile and wearable systems, a low off-state current is essential to minimize the power consumption.

For organic TFTs to have a high transit frequency, the channel length must be made as small as possible. For example, in all reports of organic TFTs with transit frequencies above 10 MHz, the channel length is below 3 μm (*9*). However, TFTs with small channel lengths often suffer from short-channel effects, such as large off-state drain currents, that result in small on/off current ratios. The key to simultaneously maximizing the transit frequency and the on/off current ratio of organic TFTs is thus to design nanoscale TFTs with a high degree of robustness against short-channel effects.

Here, we report on the design and fabrication of organic TFTs that do not show detrimental short-channel effects for channel lengths down to 300 nm, which makes it possible to simultaneously achieve an on/off current ratio of 10^10^ and a transit frequency of 40 MHz at voltages no greater than 3 V. These are the largest on/off current ratio and the highest transit frequency reported to date for flexible organic TFTs. This is an important step in the development of organic TFTs for applications that require both excellent static and excellent dynamic performance at voltages sufficiently low to be useful for mobile and wearable electronics systems.

## RESULTS

### Parameter dependencies

To better understand which parameters are limiting the on/off current ratio and the transit frequency, it is instructive to analyze the relevant equations. The drain current (*I*_D_) of a field-effect transistor operating in the linear regime can be written as follows (*10*)

$$\begin{array}{c} I_{D}=\frac{\mu_{\text{eff}}\;C_{\text{diel}}\;W}{L}\;\left[\left(V_{\text{GS}}-V_{\text{th}}\right)\;V_{\text{DS}}-\frac{V_{\text{DS}}^{2}}{2}\right]\\ \text{for}|V_{\text{GS}}-V_{\text{th}}|>|V_{\text{DS}}|\left(\text{linear regime}\right) \end{array}$$ (1)

where μ_eff_ is the effective charge-carrier mobility, *C*_diel_ is the unit-area gate-dielectric capacitance, *W* is the channel width, *L* is the channel length, *V*_GS_ is the gate-source voltage, *V*_th_ is the threshold voltage, and *V*_DS_ is the drain-source voltage. The effective charge-carrier mobility (μ_eff_) is a function of the intrinsic channel mobility (μ_0_), the contact resistance, and the channel length (*11*)

$$\mu_{\text{eff}}=\frac{\mu_{0}}{1+\frac{\mu_{0}}{L}\;R_{C}\;W\;C_{\text{diel}}\;\left(V_{\text{GS}}-V_{\text{th}}-\frac{V_{\text{DS}}}{2}\right)}$$ (2)

where *R*_C_ is the contact resistance and *R*_C_*W* is the contact resistance normalized to the channel width (*12*). Combining Eqs. 1 and 2 leads to the following equation for the drain current in the linear regime

$$\begin{array}{c} I_{D}=\frac{\mu_{0}\;C_{\text{diel}}\;W}{L+\mu_{0}\;R_{C}W\;C_{\text{diel}}\;\left(V_{\text{GS}}-V_{\text{th}}-\frac{V_{\text{DS}}}{2}\right)}\\ \left[\left(V_{\text{GS}}-V_{\text{th}}\right)\;V_{\text{DS}}-\frac{V_{\text{DS}}^{2}}{2}\right] \end{array}$$ (3)

When the channel length is sufficiently small so that the second term in the denominator of Eq. 2 is much larger than unity, Eqs. 2 and 3 can be simplified as follows

$$\mu_{\text{eff}}=\frac{L}{R_{C}W\;C_{\text{diel}}\;\left(V_{\text{GS}}-V_{\text{th}}-\frac{V_{\text{DS}}}{2}\right)}$$ (4)

$$I_{D}=\frac{W}{R_{C}W\;\left(V_{\text{GS}}-V_{\text{th}}-\frac{V_{\text{DS}}}{2}\right)}\;\left[\left(V_{\text{GS}}-V_{\text{th}}\right)\;V_{\text{DS}}-\frac{V_{\text{DS}}^{2}}{2}\right]$$ (5)

**Fig. 1.**
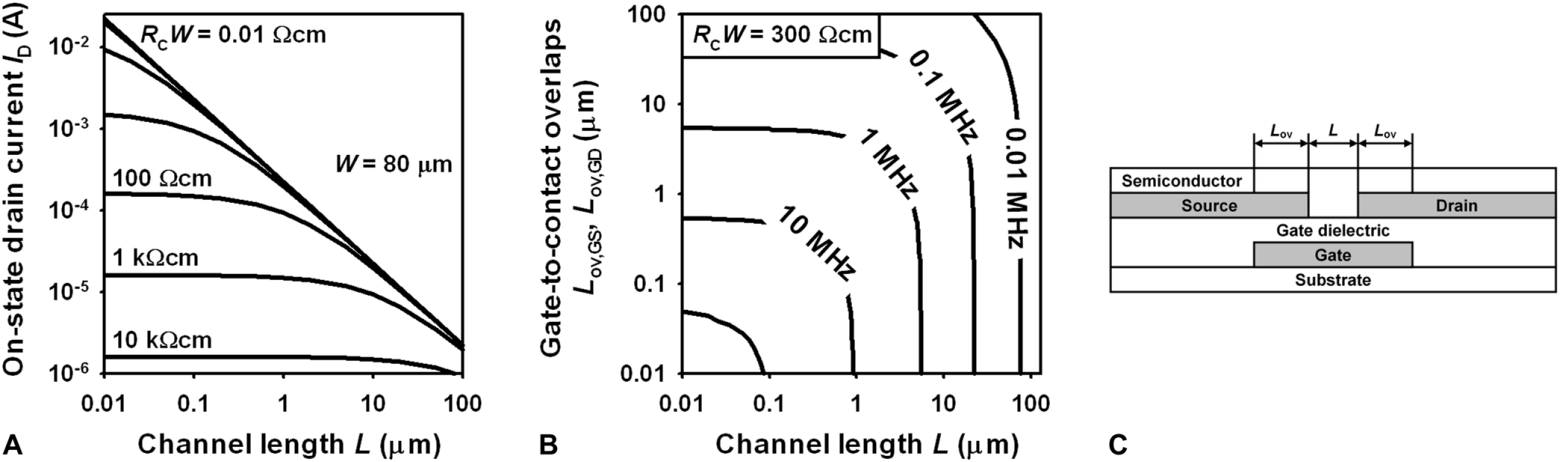
Parameter dependencies. (**A**) Dependence of the on-state drain current on the channel length and the channel width–normalized contact resistance, calculated using Eq. 3. (**B**) Dependence of the transit frequency on the channel length and the gate-to-source and gate-to-drain overlaps for a channel width–normalized contact resistance of 300 Ωcm, calculated using Eq. 7. For both graphs, the other TFT parameters were set to the following values: μ_0_ = 1 cm^2^/Vs; *C*_diel_ = 0.7 μF/cm^2^, *V*_GS_-*V*_th_ = 2 V, *V*_DS_ = 2 V, *W* = 80 μm. (**C**) Schematic cross section of a TFT showing the channel length *L* and the gate-to-source and gate-to-drain overlaps *L*_ov_.

Equation 5 indicates that for small channel lengths, the on-state drain current is independent of the channel length and is determined only by the contact resistance, the applied voltages, and the channel width. To illustrate this, solutions to Eq. 3 are plotted in Fig. 1A for channel lengths ranging from 10 nm to 100 μm and channel width–normalized contact resistances (*R*_C_*W*) ranging from 0.01 Ωcm [approximately the quantum limit of the contact resistance in field-effect transistors (*13*)] to 10 kΩcm (approximately the median value of the contact resistances reported for organic TFTs in literature). For these calculations, the other TFT parameters were set to the following values: μ_0_ = 1 cm^2^/Vs, *C*_diel_ = 0.7 μF/cm^2^, *V*_GS_-*V*_th_ = 2 V, *V*_DS_ = 2 V, *W* = 80 μm (as these are the values relevant for the TFTs reported here).

Figure 1A confirms that decreasing the channel length leads to a larger on-state drain current, as predicted by Eq. 3, but it also shows that the extent to which the drain current can be increased is limited by the contact resistance, as predicted by Eq. 5. For the contact resistance of the nanoscale TFTs reported here (300 Ωcm), the on-state drain current cannot be greater than approximately 100 μA (for a channel width of 80 μm and gate-source and drain-source voltages not exceeding 3 V). Increasing the drain current to 1 mA (for similar channel width and similar voltages) is possible only by reducing the contact resistance by an order of magnitude [see figure 5H in (*14*)]. Note that for the TFTs reported here, the second term in the denominator of Eq. 2 is on the order of 10 and therefore much larger than unity, confirming that Eqs. 4 and 5 are valid for these TFTs. Equation 5 shows that for small channel lengths, the drain current is independent not only of the channel length but also of the mobility, which means that much of the work directed toward improving the mobility of organic TFTs has little effect on the on-state drain current of nanoscale organic TFTs, since it is limited mainly by the contact resistance.

The transit frequency (*f*_T_) of a field-effect transistor operating in the linear regime can be written as follows (*15*)

$$f_{T}=\frac{\mu_{\text{eff}}\;V_{\text{DS}}}{2\;\pi \;L\;\left(L+L_{\text{ov},\text{GS}}+L_{\text{ov},\text{GD}}\right)}$$ (6)

$$f_{T}=\frac{\mu_{0}}{1+\frac{\mu_{0}}{L}\;R_{C}\;W\;C_{\text{diel}}\;\left(V_{\text{GS}}-V_{\text{th}}-\frac{V_{\text{DS}}}{2}\right)}\;\frac{V_{\text{DS}}}{2\;\pi \;L\;\left(L+L_{\text{ov},\text{GS}}+L_{\text{ov},\text{GD}}\right)}$$ (7)

where *L*_ov,GS_ and *L*_ov,GD_ are the parasitic gate-to-source and gate-to-drain overlaps (*9*). Equation 6 indicates that the transit frequency will benefit from any decrease in the critical TFT dimensions (*L*, *L*_ov,GS_, *L*_ov,GD_), regardless of how small the effective charge-carrier mobility is due to a small μ_0_, a small *L*, and/or a large *R*_C_*W*. In other words, if the on/off current ratio is irrelevant and the only goal is to maximize the transit frequency, the channel length should be made as small as possible [for example, by taking advantage of a vertical device architecture (*16*–*18*)]. However, if the goal is to maximize both the on/off current ratio and the transit frequency, there will be an optimum channel length determined by the onset of short-channel effects.

Short-channel effects occur when the ratio between the channel length and the gate-dielectric thickness is so small that the electric potential along the carrier channel is dominated by the lateral electrical field (determined by the drain-source voltage and the channel length), rather than the transverse electric field (determined by the gate-source voltage and the gate-dielectric thickness). When this happens, the undesirable injection of charges in the off-state is no longer suppressed by the transverse gate field, resulting in a larger off-state drain current and a smaller on/off ratio. To suppress short-channel effects, the ratio between the channel length and the gate-dielectric thickness must be kept above 20, ideally above 30 (*19*). For a gate-dielectric thickness of about 10 nm, this puts a lower limit of about 300 nm on the channel length. In terms of maximizing the on-state drain current, Fig. 1A shows that for a contact resistance of 300 Ωcm (which is the contact resistance of the TFTs reported here), there is little incentive for decreasing the channel length below 300 nm. Therefore, if the goal is to maximize both the on/off current ratio and the transit frequency, the optimum channel length for a gate-dielectric thickness of about 10 nm is approximately 300 nm.

**Fig. 2.**
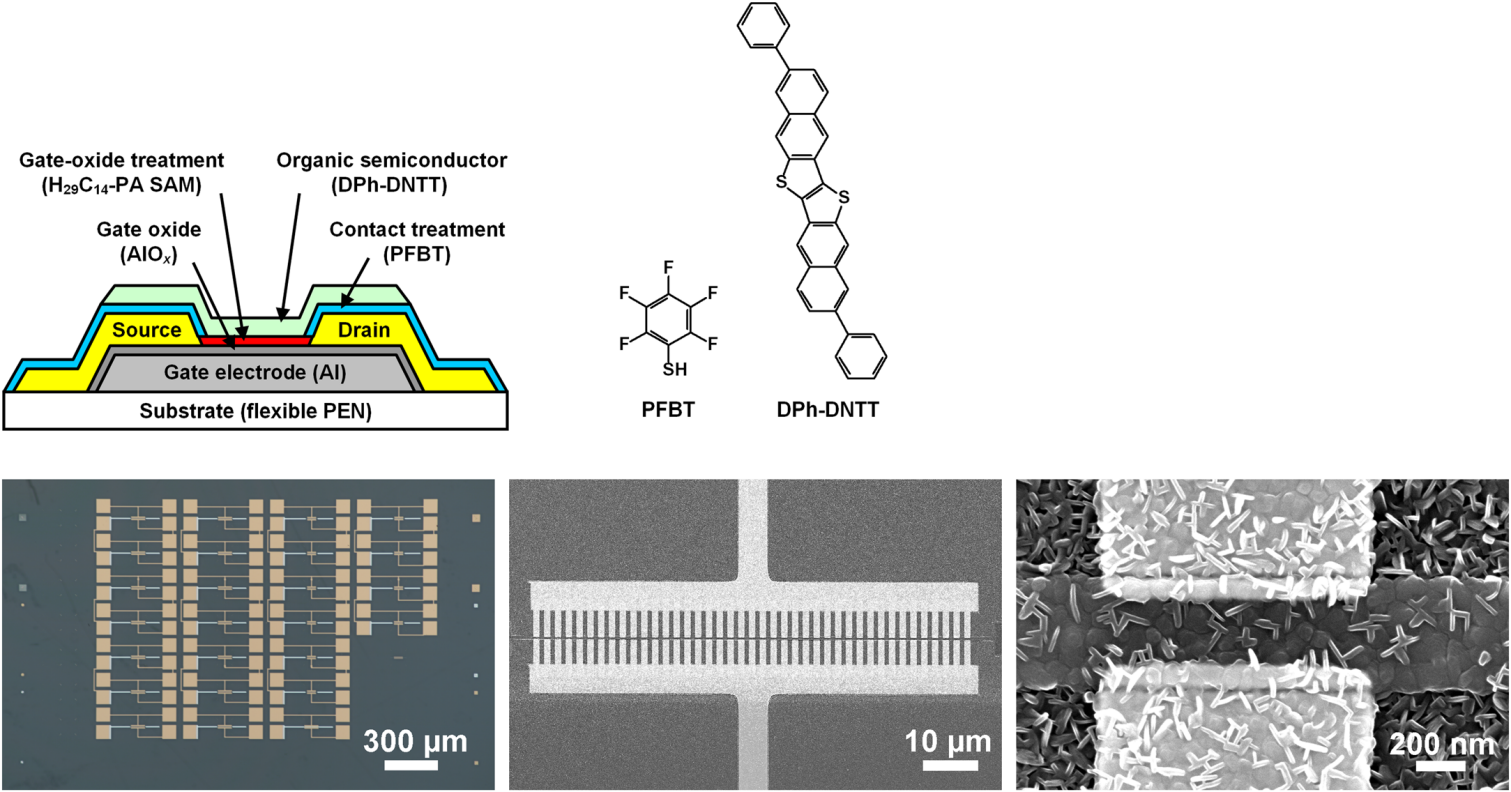
Device structure. Schematic TFT cross section, chemical structures of the molecule for the functionalization of the source and drain contacts (PFBT) and the organic semiconductor (DPh-DNTT), photograph of an array of 25 TFTs, and SEM images of a TFT with a channel length of 300 nm and gate-to-contact overlaps of 100 nm fabricated on a flexible PEN substrate.

**Fig. 3.**
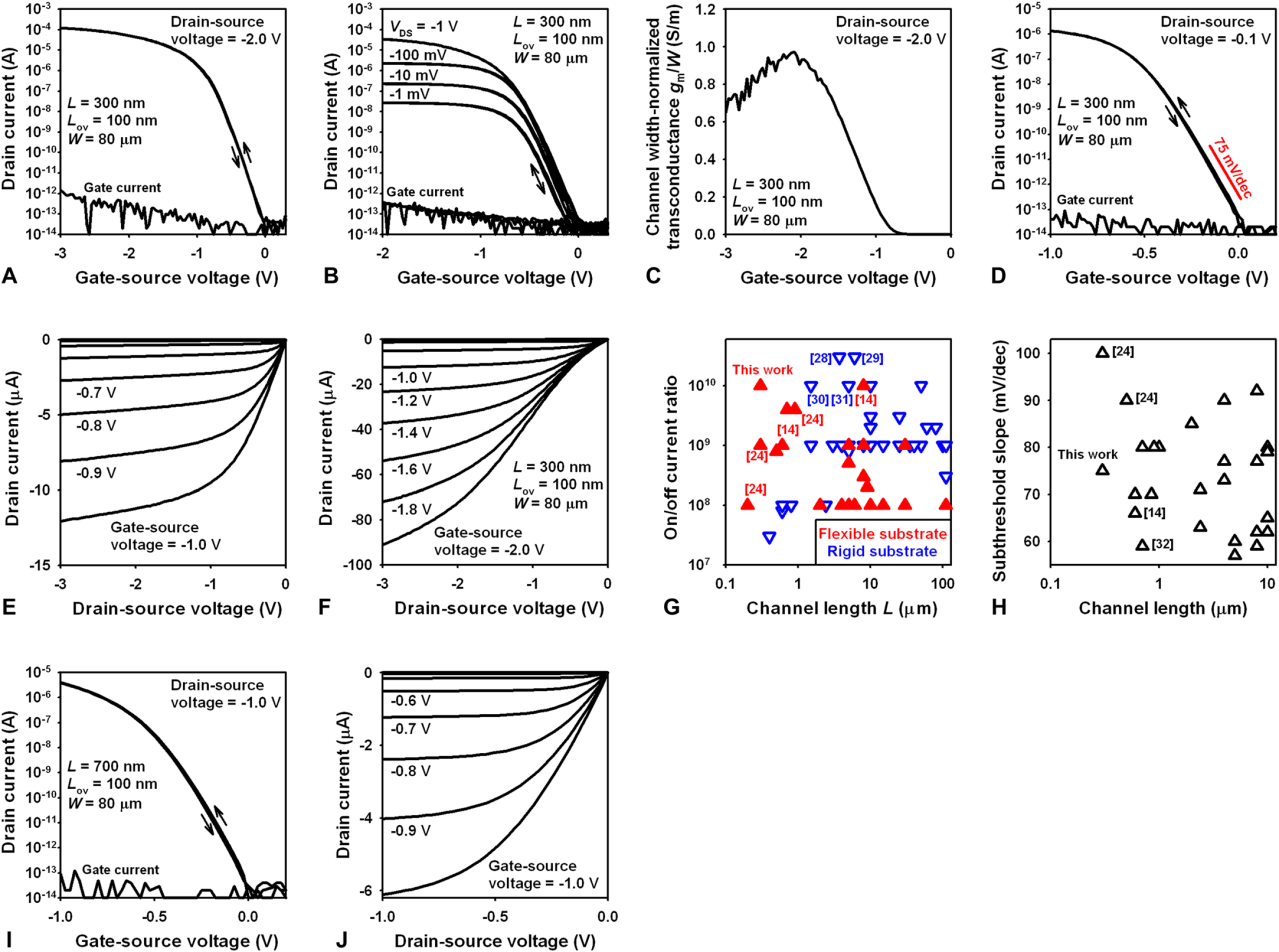
Static TFT performance. (**A** and **B**) Transfer characteristics of a TFT with a channel length of 300 nm for various drain-source voltages, indicating a turn-on voltage of 0 V and a maximum on/off current ratio of 10^10^. (**C**) Channel width–normalized transconductance (*g*_m_ = δ*I*_D_/δ*V*_GS_) calculated from the transfer characteristics. (**D**) Transfer characteristics of a TFT with a channel length of 300 nm for a drain-source voltage of −1 mV, indicating a subthreshold swing of 75 mV/decade. (**E** and **F**) Output characteristics of the same TFT for different gate-source voltages, showing good drain-current saturation for gate-source voltages up to about −1 V. (**G**) Literature summary of the on/off current ratio reported for organic TFTs with channel lengths of 100 μm or less. (**H**) Literature summary of the subthreshold swing reported for flexible organic TFTs with channel lengths of 10 μm or less. (**I**) Transfer and (**J**) output characteristics of a TFT with a channel length of 700 nm measured with gate-source and drain-source voltages no greater than −1 V, indicating an on/off current ratio of 4 × 10^8^. (*14*, *24*, *28*–*32*).

In addition to maintaining a large ratio between the channel length and the gate-dielectric thickness, it is helpful to use a semiconductor with a large HOMO (highest occupied molecular orbital)–LUMO (lowest unoccupied molecular orbital) gap. A large HOMO-LUMO gap will establish a large Schottky barrier between the Fermi level of the drain contact and the molecular orbital that is not expected to participate in charge transport (LUMO in p-channel TFTs; HOMO in n-channel TFTs). This large Schottky barrier is helpful in blocking the injection of the wrong type of charge carriers (electrons in p-channel TFTs; holes in n-channel TFTs) from the drain into the semiconductor [see figure 29 in (*20*)]. The injection of the wrong carrier type from the drain produces a large off-state drain current, making it impossible to turn the TFTs completely off; this is a well-known problem in TFTs with low-bandgap semiconductors [see figure 2E in (*21*)]. The semiconductor used for the TFTs reported here has a LUMO energy of −3.0 eV and a HOMO energy of −5.3 eV [and thus a HOMO-LUMO gap of 2.3 eV; see figure 3C in (*22*)], so assuming a work function of 5 eV for the gold source and drain contacts used here, the Schottky barrier for the undesirable injection of electrons from the drain into the LUMO has a height of approximately 2 eV. This is sufficient to keep the off-state drain current of the TFTs with *L* = 300 nm and *W* = 80 μm reported here at the noise limit of the measurement system (10 fA), but an even larger barrier height for electrons will be helpful for achieving an even better ratio between the desirable injection from the source and the undesirable injection from the drain in TFTs with smaller channel length.

Equation 6 shows that the parasitic gate-to-source and gate-to-drain overlaps (*L*_ov,GS_, *L*_ov,GD_) should be made as small as possible. This will not only maximize the transit frequency (according to Eq. 6) but also minimize the leakage currents between the gate, the source, and the drain, since these currents are approximately proportional to the overlap areas. These leakage currents unnecessarily increase both the gate current and the off-state drain current (the latter due to charge leakage from the source to the gate and from there to the drain). The TFTs reported here were fabricated by electron-beam lithography and have gate-to-contact overlaps of 100 nm. Smaller gate-to-contact overlaps will be even more desirable, because they will potentially lead to even smaller off-state leakage currents and even higher transit frequencies. With electron-beam lithography, it will certainly be possible to decrease the gate-to-contact overlaps to about 50 nm, even on flexible polymeric substrates. Even smaller overlaps may be possible with self-alignment, although it is not clear whether the backside-exposure approaches that work quite well for long-channel organic TFTs (*23*) will also work when the channel length and the gate length are on the order or smaller than the exposure wavelength.

To illustrate the dependence of the transit frequency on the critical TFT dimensions, solutions to Eq. 7 are plotted in Fig. 1B (assuming *L*_ov,GS_ = *L*_ov,GD_ and with μ_0_, *C*_diel_, *V*_GS_-*V*_th_, and *V*_DS_ being set to the same values as in Fig. 1A). Figure 1B confirms the massive incentive for decreasing the critical TFT dimensions to the nanoscale. For *L* = 300 nm and *L*_ov,GS_ = *L*_ov,GD_ = 100 nm, a transit frequency of several tens of megahertz can be expected.

### Static TFT performance

The TFTs were fabricated on flexible polyethylene naphthalate (PEN) substrates in the inverted coplanar (bottom-gate, bottom-contact) device architecture. Aluminum gate electrodes and gold source and drain contacts were patterned by electron-beam lithography (*24*). The gate dielectric is a stack of plasma-grown aluminum oxide (AlO*_x_*) and a self-assembled monolayer (SAM) of *n*-tetradecylphosphonic acid (H_29_C_14_-PA) with a total thickness of 8 nm and a unit-area capacitance of 0.7 μF/cm^2^ (*25*). Before the deposition of the organic semiconductor [vacuum-deposited 2,9-diphenyl-dinaphtho[2,3-b:2′,3′-f]thieno[3,2-b]thiophene (DPh-DNTT) (*26*)], the surface of the source and drain contacts was functionalized with a chemisorbed monolayer of pentafluorobenzenethiol (PFBT) to minimize the contact resistance (*27*). A schematic cross section of the TFTs, the chemical structures of PFBT and DPh-DNTT, and a photograph and two scanning electron microscopy (SEM) images of the TFTs are shown in Fig. 2.

The static performance of the TFTs is summarized in Fig. 3. The channel width is 80 μm. For a channel length of 300 nm, the TFTs have a turn-on voltage of 0 V, on-state and off-state drain currents of 100 μA (at *V*_GS_ = −3 V) and 10 fA (at *V*_GS_ = 0), an on/off current ratio of 10^10^, a channel width–normalized transconductance of 1 S/m, and a subthreshold swing of 75 mV/decade (Fig. 3, A to F). The on/off current ratio (10^10^) is the largest reported to date for flexible organic TFTs, and the largest reported for organic TFTs with a channel length below 1 μm [Fig. 3G; (*28*–*31*)]. The subthreshold swing (75 mV/decade) is the smallest reported for flexible organic TFTs with a channel length below 600 nm [Fig. 3H; (*32*)]. The off-state drain current is at the noise limit of the measurement setup (10 fA). Despite the fact that the thickness of the gate dielectric is less than 10 nm, the gate current of the TFTs is below 1 pA.

The fact that the turn-on voltage is exactly 0 V is highly desirable, because it makes it possible to achieve a large on/off current ratio with minimum applied voltages. To illustrate this, Fig. 3 (I and J) shows the transfer and output characteristics of a TFT with a channel length of 700 nm operated with gate-source and drain-source voltages no greater than −1 V. The transfer characteristics indicate an on/off current ratio of 4 × 10^8^, which is the largest on/off current ratio reported to date for organic TFTs over a gate-source-voltage range from 0 to ±1 V or less. Figure S1 summarizes the air stability and device-to-device uniformity of the TFTs.

**Fig. 4.**
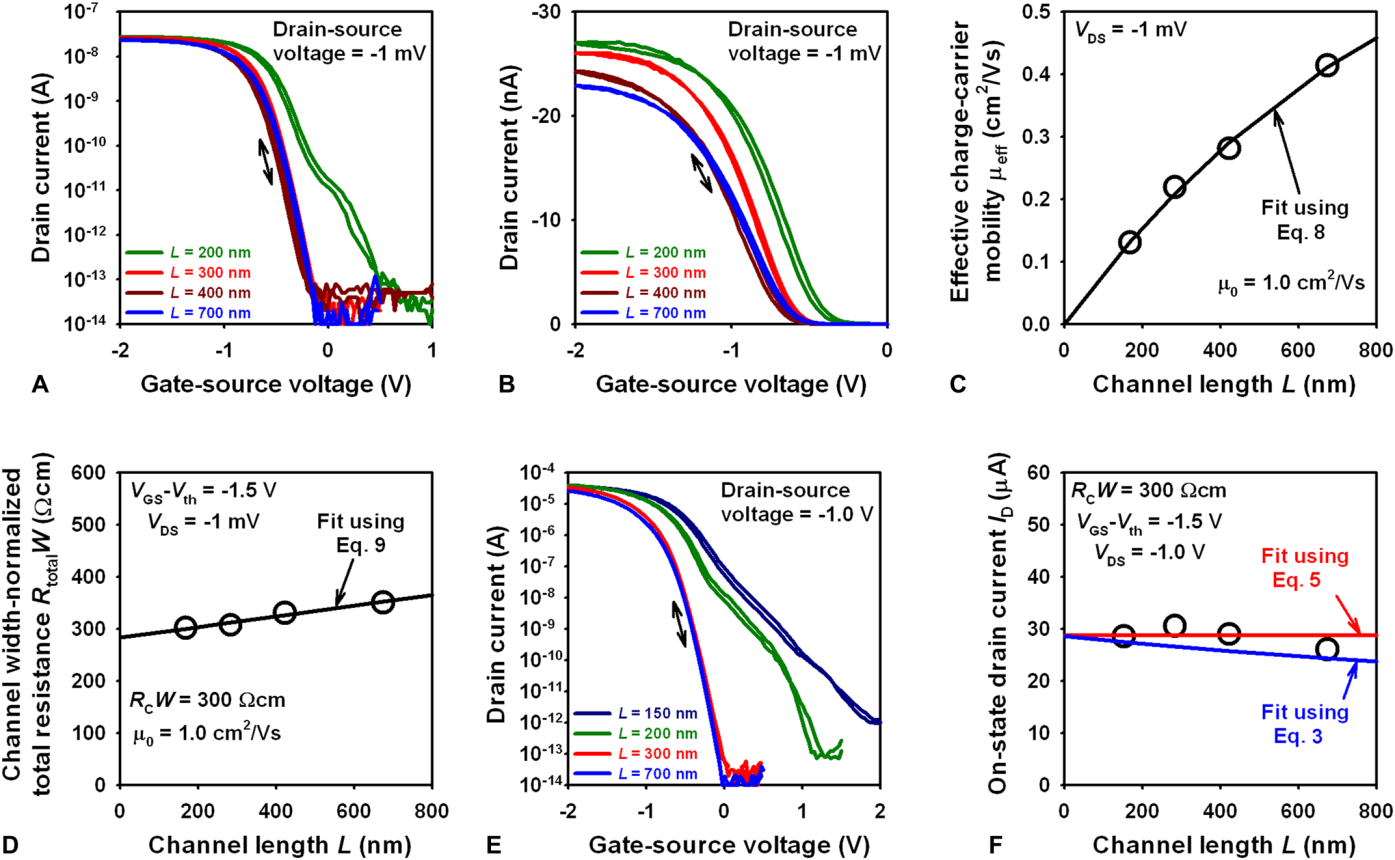
Channel-length dependence of the static TFT characteristics. (**A** and **B**) Transfer characteristics of TFTs with channel lengths ranging from 200 to 700 nm measured at a drain-source voltage of −1 mV. (**C**) Effective charge-carrier mobilities (μ_eff_) extracted from the linear regions of the transfer curves plotted versus the channel length *L*. From this dependence of μ_eff_ on *L*, an intrinsic channel mobility (μ_0_) of 1 cm^2^/Vs is extracted. (**D**) Transmission line method, yielding a contact resistance of 300 Ωcm. (**E**) Transfer characteristics of TFTs with channel lengths ranging from 150 to 700 nm measured at a drain-source voltage of −1 V, illustrating the onset of short-channel effects for *L* < 300 nm. (**F**) On-state drain currents plotted as a function of channel length, with fit lines calculated using Eqs. 3 and 5.

Figure 4 (A and B) shows the transfer characteristics of TFTs with channel lengths ranging from 200 to 700 nm measured at a drain-source voltage of −1 mV. The effective charge-carrier mobilities (μ_eff_) extracted from the linear regions of these transfer curves (extracted by fitting Eq. 1 to the measurement data) range from 0.1 cm^2^/Vs for *L* = 200 nm to 0.4 cm^2^/Vs for *L* = 700 nm (Fig. 4C). From this dependence of μ_eff_ on *L*, the intrinsic channel mobility μ_0_ is obtained by fitting the following equation (*27*)

$$\mu_{\text{eff}}=\frac{\mu_{0}}{1+\frac{L_{1/2}}{L}}$$ (8)

where *L*_1/2_ is a characteristic channel length at which the contact resistance equals the channel resistance. From the fit, the intrinsic channel mobility (μ_0_) is determined to be 1 cm^2^/Vs (Fig. 4C). The channel width–normalized contact resistance (*R*_C_*W*) is estimated by applying the transmission line method (TLM) to the transfer curves using the following equation (*27*)

$$R_{\text{total}}W=R_{C}W+\frac{L}{\mu_{0}\;C_{\text{diel}}\;\left(V_{\text{GS}}-V_{\text{th}}\right)}$$ (9)

where *R*_total_*W* is the channel width–normalized total device resistance, calculated from the measurement data as *R*_total_ = *V*_DS_/*I*_D_. By fitting Eq. 9 to the *R*_total_*W* versus *L* data, the contact resistance is determined to be 300 Ωcm, and the intrinsic channel mobility μ_0_ is determined to be 1 cm^2^/Vs (Fig. 4D). To our knowledge, the TLM has not been applied previously to submicrometer-channel-length organic TFTs. [In (*27*), it was shown that the reliability of the TLM benefits from being performed on TFTs with small channel lengths.]

Figure 4E shows the transfer characteristics of TFTs with channel lengths ranging from 150 to 700 nm measured at a drain-source voltage of −1 V. Unlike the TFTs with channel lengths of 300 nm or above, which are characterized by a complete lack of detrimental short-channel effects, the TFTs that have channel lengths below 300 nm are characterized by pronounced short-channel effects, including larger off-state drain currents and smaller on/off current ratios (3 × 10^8^ for *L* = 200 nm; 3 × 10^7^ for *L* = 150 nm). Although these are still the largest on/off ratios reported for organic TFTs with such small channel lengths, they are smaller than the on/off ratio of 10^10^ obtained for a channel length 300 nm, confirming that for the TFT technology used here (in particular, for a gate-dielectric thickness of 8 nm), 300 nm is the minimum channel length required to suppress short-channel effects and obtain the largest possible on/off current ratio. In Fig. 4F, the experimentally determined on-state drain currents are plotted versus the channel length, and as can be seen, they are in good agreement with the fit lines calculated using Eqs. 3 and 5, confirming that Eq. 5 is valid for the nanoscale TFTs reported here.

### Dynamic TFT performance

**Fig. 5.**
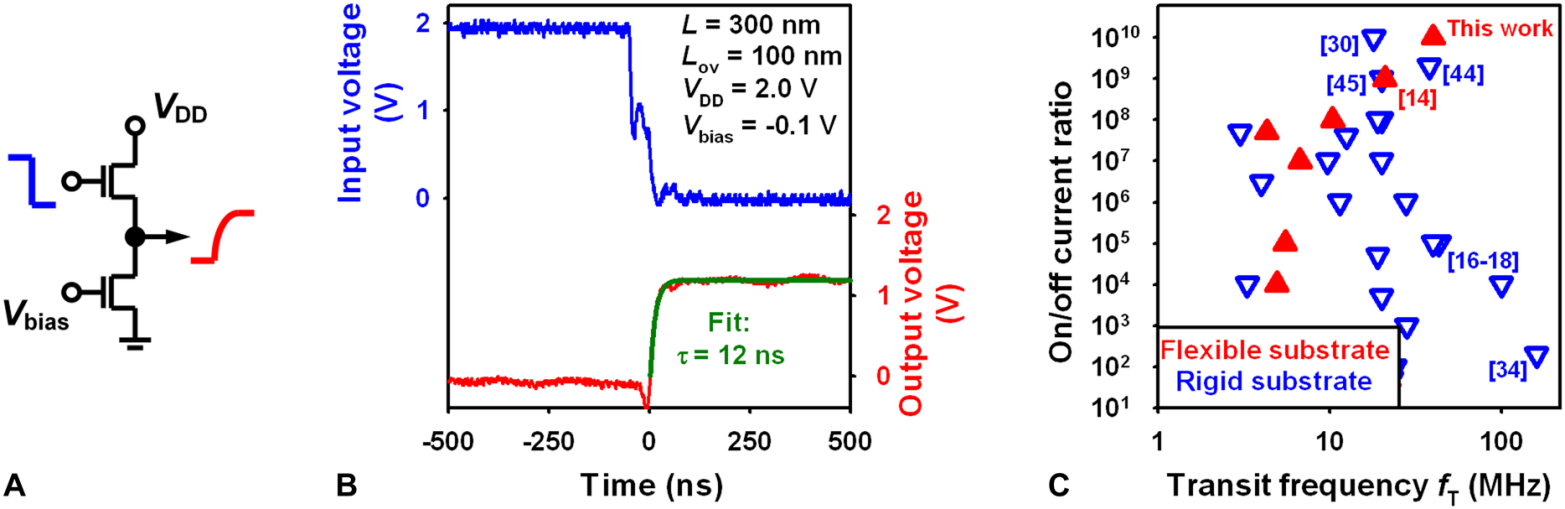
Dynamic TFT performance. (**A**) Setup for measuring the dynamic switching response of a biased-load inverter. (**B**) Dynamic switching response of an inverter based on two TFTs with a channel length of 300 nm and gate-to-source and gate-to-drain overlaps of 100 nm. (Note that the voltage spike in the falling edge of the input signal is caused by the displacement current that flows into or out of the inverter’s input node during switching; see Fig. S2.) (**C**) Literature summary of the on/off current ratio reported for organic TFTs with transit frequencies greater than 1 MHz (*14*, *16*–*18*, *30*, *34*, *44*, *45*).

To evaluate the dynamic performance of the nanoscale organic TFTs, we fabricated inverters based on two transistors, each having a channel length of 300 nm and gate-to-contact overlaps of 100 nm. The inverters were designed in the biased-load circuit topology (*33*) and fabricated on a flexible PEN substrate. A square-wave voltage with an amplitude of 2 V was applied to the input of the inverter using a waveform generator, and the inverter’s output response was recorded using a high-impedance probe and an oscilloscope (Fig. 5A). Fitting an exponential function to the measured output signal yields a characteristic switching-delay time constant (τ) of 12 ns at a supply voltage (*V*_DD_) of 2 V (Fig. 5B). This switching-delay time constant corresponds to a transit frequency [*f*_T_ = 1/(2τ)] of 40 MHz and a supply voltage–normalized transit frequency (*f*_T_/*V*_DD_) of 20 MHz/V. These are the highest transit frequency and the highest voltage-normalized transit frequency reported to date for flexible organic TFTs.

More importantly though, this record dynamic performance does not come at the expense of poor static performance, as the nanoscale TFTs reported here simultaneously exhibit both excellent static and excellent dynamic performance, exemplified by the highest on/off current ratio (10^10^) and the highest transit frequency (40 MHz) reported for flexible organic transistors (Fig. 5C). This is a critical advance in the development of high-performance organic TFTs. There are only five reports of organic TFTs with higher transit frequencies (*16*–*18*, *30*, *34*), and all of them were fabricated on rigid substrates and operated with higher voltages. In four of these five reports, the TFTs have on/off current ratios no greater than 10^5^. Sawada *et al.* (*30*) reported TFTs with transit frequencies between 10 MHz (for *L* = 10 μm) and 45 MHz (for *L* = 1.5 μm) at 7 V and an on/off ratio of 10^10^ for *L* = 6 μm [the on/off ratio of the TFTs with *L* ≤ 6 μm and *f*_T_ ≥ 20 MHz is not mentioned in (*30*)].

## DISCUSSION

### Contact resistance and on-state drain current

Equation 5 shows that for a fixed channel width and fixed voltages, the on-state drain current can be improved further only by decreasing the contact resistance. Although the contact resistance of the TFTs reported here (300 Ωcm) is already smaller than the contact resistance of 90% of all organic TFTs reported in literature (750 reports; median: 17 kΩcm; average: 2.9 MΩcm), further improvements are certainly feasible. For example, contact resistances ranging from 1 to 30 Ωcm have been reported for organic TFTs in which the source and drain contacts were prepared by stencil lithography (*14*, *27*, *32*) or mechanical transfer of van der Waal contacts (*35*, *36*). The problem is that these methods are currently not capable of producing sufficiently small gate-to-source and gate-to-drain overlaps. Figure 1B shows that for transit frequencies above 10 MHz, not only the channel length but also the gate-to-contact overlaps must be below 1 μm. Stencil lithography and mechanical transfer are capable of producing channel lengths as small as 300 nm (*37*) and 1.5 μm (*36*), respectively, but neither method is capable of producing gate-to-contact overlaps below 1 μm. One of the key challenges for future work will thus be to reduce the contact resistance of organic TFTs with nanoscale channel lengths and nanoscale gate-to-contact overlaps. Figure 1A shows that reducing the contact resistance of nanoscale organic TFTs by an order of magnitude will lead to an increase in on-state drain current (and thus to an increase in on/off current ratio) by an order of magnitude (and also to an increase in transit frequency). This is a massive incentive for intensifying the efforts to reduce the contact resistance of nanoscale organic TFTs. The main obstacle that needs to be overcome in this quest is the Fermi-level pinning by metal-induced gap states (*38*–*40*).

### Channel width and on/off current ratio

According to Eq. 5, the on-state drain current is proportional to the channel width (*W*). While all the TFTs reported here have a channel width of 80 μm, it is certainly possible to fabricate TFTs with larger channel widths. However, it is likely that increasing the channel width will increase not only the on-state drain current but also the off-state drain current, probably at a similar rate. Therefore, as soon as the off-state drain current is above the noise limit of the measurement setup (10 fA), a larger channel width will not lead to a larger on/off current ratio, unless additional measures are taken to further suppress the off-state currents. Two such measures have already been mentioned: a semiconductor with a larger HOMO-LUMO gap (to establish a larger energy barrier against the undesirable injection of the wrong carrier type from the drain into the semiconductor) and further reductions of the gate-to-contact overlaps (to minimize the leakage currents through the gate dielectric). The latter approach provides the additional benefit of also leading to a higher transit frequency, as indicated by Eq. 6.

### Channel length and gate-dielectric thickness

Figure 4E shows that short-channel effects dramatically reduce the on/off current ratio. To be able to decrease the channel length (with the goal of increasing the transit frequency) without compromising the on/off current ratio therefore requires a concurrent reduction of the gate-dielectric thickness to keep the ratio between the channel length and the gate-dielectric thickness above 20, ideally above 30 (*19*). While the gate-dielectric thickness of the TFTs reported here (8 nm) is already smaller than that of most organic TFTs reported in literature, further reductions of the gate-dielectric thickness are certainly feasible [see, e.g., figure 1 in (*25*)]. As in silicon microelectronics, the main challenge will be to suppress the gate leakage, especially once the probability for quantum-mechanical tunneling becomes large. This is true regardless of whether the TFTs are designed in a lateral device architecture (as shown here) or in a vertical device architecture (*16*–*18*). Decreasing the gate-to-contact overlaps will be beneficial here as well, since the gate current is proportional to the overlap area. The ultimate goal is a TFT with a gate-dielectric thickness of approximately 3 to 5 nm, a channel length of approximately 100 to 150 nm, and near-zero gate-to-contact overlaps.

## MATERIALS AND METHODS

### Substrates and device architecture

The TFTs were fabricated in the bottom-gate, bottom-contact (inverted coplanar) device architecture on a 125-μm-thick flexible PEN substrate (Teonex Q65 PEN).

### Gate electrodes and gate dielectric

To define the gate electrodes, the substrate was coated with a two-layer resist consisting of a bottom layer of poly(methyl methacrylate) (PMMA) having a molecular weight of 200k and a thickness of 200 nm (Allresist AR-P 641.05) and a top layer of PMMA having a molecular weight of 950k and a thickness of 120 nm (Allresist AR-P 671.025). The purpose of using a two-layer resist is to create a reentrant (undercut) profile to enable clean lift-off of the excess metal. Electron-beam lithography was performed using a Raith eLINE (electron-beam voltage 20 kV, exposure dose 370 μC/cm^2^). The resist patterns were developed in a 2-propanol solution of methyl isobutyl ketone (MIBK). Aluminum with a thickness of 30 nm was deposited by thermal evaporation in vacuum (background pressure 10^−7^ mbar, deposition rate 2 nm/s); this layer has a surface roughness of about 1 nm (*41*). The aluminum surface was exposed to oxygen plasma [oxygen flow rate 30 sccm (standard cubic centimeter per minute), oxygen partial pressure 10 mTorr, plasma power 200 W, plasma duration 60 s] to produce an aluminum oxide (AlO*_x_*) gate dielectric on the surface of the Al gate electrodes with a thickness of about 6 nm (*25*). Patterning of the gate electrodes was completed by lifting off the excess metal in *N*-ethyl-2-pyrrolidone (NEP; Allresist Remover AR 300-70).

### Source and drain contacts

To define the source and drain contacts, the substrate was again coated with a two-layer PMMA resist, followed by electron-beam lithography and development in MIBK. Titanium with a thickness of 0.3 nm (to improve adhesion) and gold with a thickness of 30 nm were sequentially deposited by thermal evaporation in vacuum (background pressure 10^−7^ mbar, deposition rate 0.03 nm/s). Patterning of the source and drain contacts was completed by lift-off in NEP.

### Monolayer functionalization of the gate dielectric and the source/drain contacts

To promote a favorable thin-film morphology of the organic semiconductor in the channel region and on the surface of the source/drain contacts (*42*), the substrate was immersed into a mixed 2-propanol solution of *n*-tetradecylphosphonic acid (H_29_C_14_-PA; PCI Synthesis, Newburyport, MA, USA) (*43*) and PFBT (TCI Germany). The *n*-tetradecylphosphonic acid forms a hydrophobic SAM on the surface of the AlO*_x_* gate dielectric with a thickness of approximately 2 nm, and the hybrid AlO*_x_*/SAM gate dielectric that then separates the organic-semiconductor layer from the gate electrodes has a total thickness of 8 nm and a unit-area capacitance of 0.7 μF/cm^2^ (*25*). The PFBT forms a chemisorbed monolayer on the surface of the Au source and drain contacts that helps to minimize the contact resistance of the TFTs (*42*).

### Organic semiconductor

In the last process step, the small-molecule semiconductor 2,9-diphenyl-dinaphtho[2,3-b:2′,3′-f]thieno[3,2-b]thiophene [DPh-DNTT (*26*); Nippon Kayaku; provided by K. Ikeda, Y. Sadamitsu, and S. Inoue] was deposited by thermal sublimation in vacuum (background pressure 10^−7^ mbar, deposition rate 0.03 nm/s) through a manually aligned stencil mask (*9*). During the organic-semiconductor deposition, the substrate was held at a temperature of 90°C.

### Characterization

All electrical measurements were performed in ambient air at room temperature using a Micromanipulator 6200 manual probe station. The static output and transfer characteristics of the TFTs were recorded using an Agilent 4156C Semiconductor Parameter Analyzer. The connections between the parameter analyzer and the probe needles were made using Kelvin triaxial cables, and the measurements were carried out with an integration time of 20 ms; in this configuration, the measurement noise floor is 10 fA, limited by leakage in the triaxial cables. The dynamic characteristics of the inverters were recorded using a Keysight 33622A Waveform Generator, a Textronix TDS 1001B Digital Oscilloscope, and a GGB Industries Model 19C Picoprobe.
