# Supplementary material for: Flexible low-voltage organic transistors with a transit frequency of 40 MHz and an on/off current ratio of 10 orders of magnitude
Source: Sci Adv. 2025 Dec 17;11(51):eaeb9693. doi: 10.1126/sciadv.aeb9693 (PMC12710694; doi:10.1126/sciadv.aeb9693)
Supplement: Supplementary file 1 — Figs. S1 and S2 [file sciadv.aeb9693_sm.pdf]

Science Advances
AAAS

Supplementary Materials for

Flexible low-voltage organic transistors with a transit frequency of 40 MHz and an on/off current ratio of 10 orders of magnitude

Ute Zschieschang and Hagen Klauk

Corresponding author: Hagen Klauk, h.klauk@fkf.mpg.de

Sci. Adv. **11**, eaeb9693 (2025)
DOI: 10.1126/sciadv.aeb9693

This PDF file includes:

Figs. S1 and S2

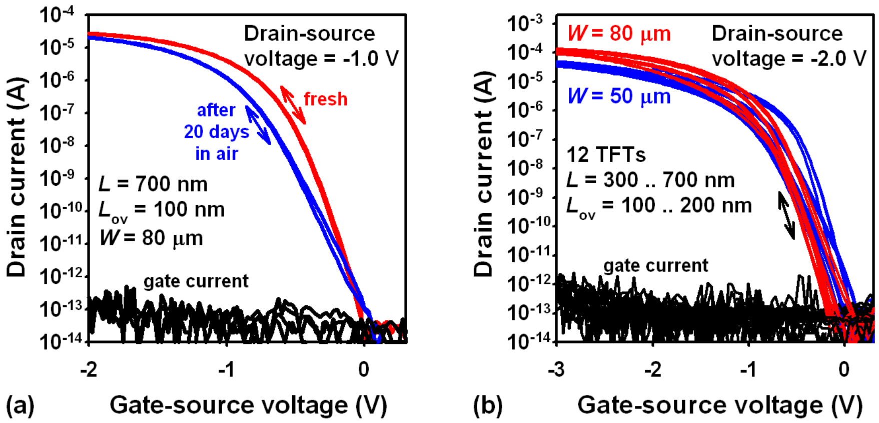

Fig. S1. Air stability and device-to-device uniformity. (a) Transfer characteristics of a TFT with a channel length of 700 nm measured immediately after device fabrication (red curve) and measured again after 20 days of storage in ambient air (blue curve). (b) Transfer characteristics of 12 TFTs with channel lengths ranging from 300 to 700 nm fabricated over a period of four years. All TFTs have turn-on voltages close to 0 V, on/off current ratios above 10^8, and transit frequencies greater than 10 MHz (estimated using Equation 7).

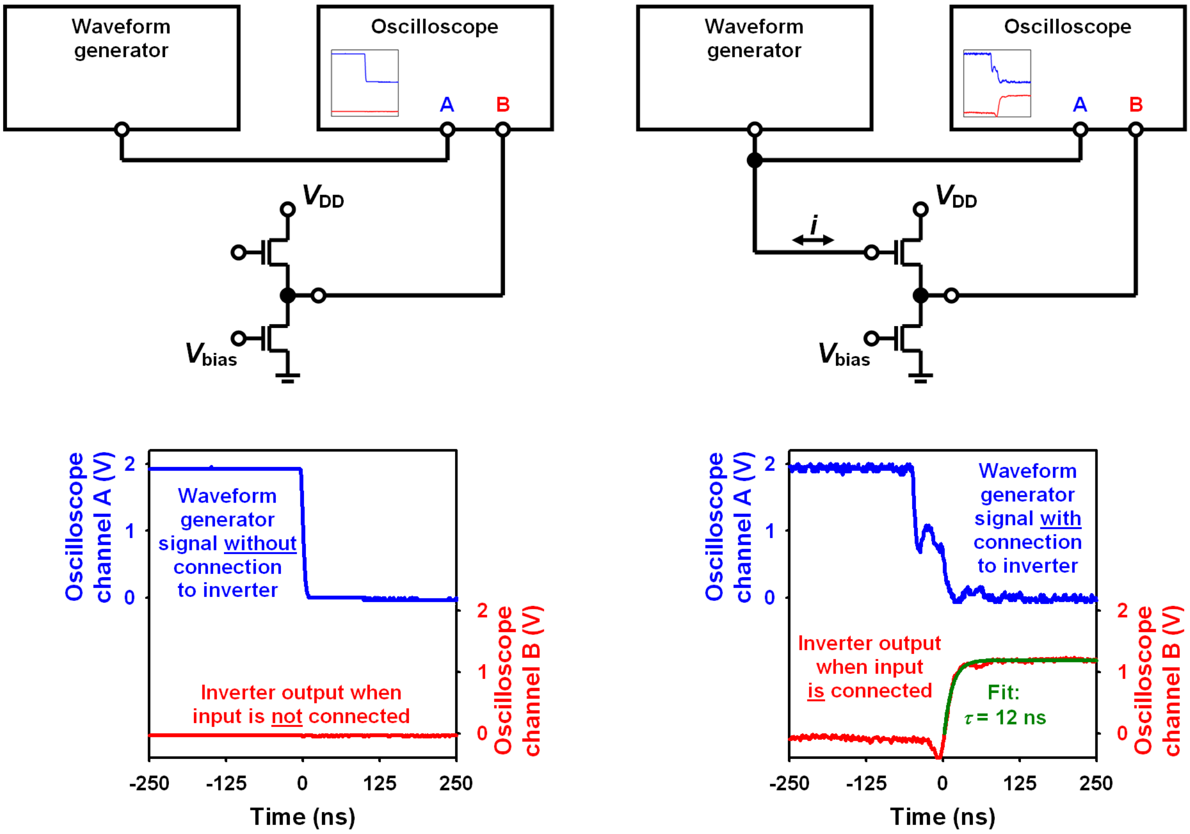

Fig. S2. Inverter measurement. Circuit diagrams and oscilloscope signals illustrating the measurement of the dynamic switching response of the inverters and the appearance of a voltage spike in the output signal of the waveform generator when the waveform generator is connected to the input node of the inverter.

Left: When the output terminal of the waveform generator is not connected to the input node of the inverter, the output signal of the waveform generator appears as a clean step function on the oscilloscope.

Right: When the output terminal of the waveform generator is connected to the input node of the inverter, a voltage spike appears in the output signal of the waveform generator. This is caused by the displacement current that flows into or out of the inverter's input node, i.e., into or out of the gate electrode of the drive TFT of the inverter, in response to changes of the voltage applied to the inverter's input node, such as those initiated by the output signal of the waveform generator: $i = \delta q/\delta t = C{\cdot}\delta v/\delta t$, where i is the displacement current, q is the charge on the gate electrode of the drive TFT, C is the gate capacitance of the drive TFT, v is the inverter's input voltage, and t is the time. For the measurement setup employed here, $C = 0.3$ pF ($C = C_{\text{diel}}(L{+}2{\cdot}\Delta L)W$; $C_{\text{diel}} = 0.7$ µF/cm^2, $L = 300$ nm, $\Delta L = 100$ nm, $W = 80$ µm), $\delta v = 2$ V, and $\delta t = 3$ ns (rise and fall times of the Keysight 33622A Waveform Generator). For these parameter values, the displacement current i reaches a magnitude of 0.2 mA, which causes the appearance of a voltage spike in the output signal of the waveform generator.
